# Can Hepatitis C Virus Antigen Testing Replace Ribonucleic Acid Polymearse Chain Reaction Analysis for Detecting Hepatitis C Virus? A Systematic Review

**DOI:** 10.1093/ofid/ofw252

**Published:** 2017-05-26

**Authors:** Harun Khan, Andrew Hill, Janice Main, Ashley Brown, Graham Cooke

**Affiliations:** 1 Faculty of Medicine, Imperial College London, United Kingdom; 2 Department of Pharmacology and Therapeutics, Liverpool University, United Kingdom

**Keywords:** HCV antigen, HCV diagnostics, HCV RNA

## Abstract

The complexity and cost of current diagnostics for hepatitis C virus (HCV) may act as a prevention to the scale-up of treatment in the developing world. Currently, ribonucleic acid (RNA)-polymerase chain reaction tests are the gold standard. However, there is potential for the use of simpler and cheaper antigen tests to confirm HCV infection in different clinical settings. We evaluated the sensitivity and specificity of antigen assays. This was compared with the reference-standard RNA assays. A subanalysis also assessed Architect core antigen test, which is the only commercially available antigen test on the market. In 24 datasets, evaluating HCV-antigen assays in 8136 samples, the percentage of HCV-antigen positive, HCV-RNA negative was 0.57%. The percentage HCV-antigen negative, HCV-RNA positive was 3.52%. There is strong evidence that antigen detection performs as well as RNA-based assays for HCV management. The use of antigen tests could improve access to HCV care in underresourced healthcare settings.

Although treatments for hepatitis C virus (HCV) are becoming simpler and more effective, the complexity and high cost of current diagnostics have the potential to act as obstacles to the scale-up of treatment in resource-poor settings [1], even with the advent of treatments with pangenotypic activity.

Anti-HCV tests are typically used to screen for exposure to virus with a more complex and expensive ribonucleic acid (RNA) amplification test (HCV-RNA polymerase chain reaction [PCR]) to confirm viremia and stratify treatment decisions. However, because viral kinetics no longer predict treatment duration or outcome, there is no longer a need for accurate quantitative load measurement. There remains an unmet need for a cheap, simple test to differentiate those with ongoing viral replication (who therefore require treatment) from those who have previously been exposed but have spontaneously cleared the virus [1] and to confirm successful eradication after antiviral therapy.

An alternative, simpler approach to confirm viremia is viral antigen detection. One test (Abbott Architect HCV core antigen [cAg] assay) is already commercially available. Newer antigen tests currently under development are likely to need less laboratory support and could be performed closer to the point of care (POC) [2]. However, before antigen testing can be widely adopted, evidence is needed to confirm the performance of antigen testing against HCV-RNA PCR (currently considered a gold standard), in different clinical settings.

We conducted a systematic review of the accuracy of antigen assays for HCV in different populations using HCV-RNA PCR as a reference standard, calculating the percentage of false-positive (FP) and false-negative (FN) values. A subanalysis also assessed the sensitivity and specificity of the Abbott Architect cAg.

## METHODS

### Data Search and Selection

This review adhered to the PRISMA guidelines for reporting systematic reviews [3]. It aimed to include all publications that evaluated the detective accuracy of antigen tests for HCV in any population—using RNA assays as a reference standard.

Two databases (EMBASE and MEDLINE) were used for the original search. Both free-text and Medical Subject Headings (MeSH) terms were used as search items (Supplementary Table S1). An initial search was conducted to ascertain the most appropriate terms to use.

The following limits were applied: availability of an English-language translation and publication date. All literature was published between January 1, 2004 and May 14, 2015. A pilot search from 2003 showed 88 relevant papers—many did not identify the assays that were used, hence these were excluded. Reference lists were also searched. All extracted literature was screened, independently, by 2 authors to assess eligibility for inclusion. After the initial screening process, all publications were assessed for eligibility based on their full-texts.

### Inclusion and Exclusion Criteria

Studies were either prospective, cross-sectional, cohort, or retrospective, case-control studies by design. They compared HCV-detection results from one sample of patients who were tested with an antigen assay and a nucleic acid amplification assay—the latter was always used as a reference standard. Articles were excluded if they did not cite the name or manufacturer of the assay, because this would not allow for an assay-specific evaluation of detective accuracy. Articles that did not present the raw discordance data were excluded, because this would affect the accuracy of our analyses.

### Outcomes

The primary outcomes for all publications—independent of study design—were as follows: (1) the percentage FP, which was defined as the percentage of antigen-positive, RNA-negative results amongst all tests; and (2) the percentage FN, which was defined as the percentage of antigen-negative, RNA-positive results amongst all tests. These analyses were replicated for 8 studies that evaluated Architect cAg assay, Cobas AmpliPrep/COBAS TaqMan (CACTM), which was used as a reference standard in these studies. There were no secondary outcomes of interest.

### Data Screening and Extraction

Publications that reflected our main objectives were reviewed, and relevant data were extracted by the first reviewer (H. K.) and checked by the second (A. H.). In cases of disagreement, a third, independent reviewer was consulted (G. C.). Data extraction included the following: basic study information [author(s), publication date, country]; population data (sample size and description); assay profile (name ± manufacturer, lower limit of detection [LLOD]); and HCV-detection results (number of HCV-positive and HCV-negative patients using antigen and RNA assays).

The LLOD of RNA tests are traditionally expressed in international units per milliliter (IU/mL), whereas the LLOD of antigen tests is expressed in alternative units (fmol/L or pg/mL). For ease of comparison, our review expressed the LLOD, for both antigen and RNA tests, in IU/mL. When the LLOD was not clarified, it was cross-referenced using the aforementioned UNITAID report [2].

### Statistical Analysis

We calculated the specificity and sensitivity (presented as percentage-format) using raw data from each individual publication. Each publication was grouped by manufacturer and LLOD (±100 IU/mL) (Table 1). Publications with less common assays and/or with nonstandard LLODs were not allocated to a group. Weighted averages of sensitivity and specificity were calculated for each group (and each individual nonassigned study) and then combined in an overall weighted average. Confidence intervals ([CIs] 95%) were provided for each statistic. Group 1 formed a subanalysis. A receiver operator characteristic (ROC) curve was plotted using specificity and sensitivity data of all publications. This is a recommended method to present diagnostic studies data [4] and may confirm or disprove a relationship between diagnostic tests. This was not undertaken for the subanalysis. A strict meta-analysis was not conducted because of the heterogeneity amongst HCV-detection assay models.

**Table 1.** Categorization of Publications for Analysis

Subgroup	Antigen Assay (Manufacturer)	RNA Assay (Manufacturer)
Group 1	Architect cAg (Abbott)	CACTM (Roche)
Group 2	Architect cAg (Abbott)	(Abbott)
Group 3a	(Ortho—low LLOD)	(Roche)
Group 3b	(Ortho—high LLOD)	(Roche)
Group 4	(Ortho)	(Versant)
Other	–	–

Abbreviations: CACTM, Cobas AmpliPrep/COBAS TaqMan; cAg, core antigen; LLOD, lower limit of detection; RNA, ribonucleic acid.

### Quality Assessment

The QUADAS-2 tool was used to assess the quality of all included studies [5]. It is designed specifically for primary diagnostic studies. It assessed studies in 4 areas: patient selection process, the test being studied (antigen assay), the reference standard (RNA assay), and patient progression in the study (eg, timing of tests). Each area was evaluated in respect to their “risk of bias”. The first 3 areas were evaluated for applicability too. The quality assessment was solely used to exclude low quality studies retrospectively. It was summarized in Supplementary Figures S1 and S2.

## RESULTS

### Study Characteristics

Of 878 papers screened, 4 were identified through an independent contact and no articles were found through searching bibliographies—263 abstracts were assessed. Subsequently, 47 publications were assessed for eligibility in full-text format. An initial review excluded 24 publications. Ultimately, 23 publications were eligible for use in statistical analysis (according to inclusion and exclusion criteria). One study compared the same antigen assay against 2 different reference standards [6]—both data sets were included to form 24 datasets in total.

Eighteen studies were prospective, cross-sectional cohort studies, whereas 6 were retrospective, case-control studies. Eight studies evaluated Architect cAg, using CACTM as a reference RNA standard (Group 1). The paper quality was rated as high. This was most likely attributed to the strict eligibility criteria.

**Figure 1.**
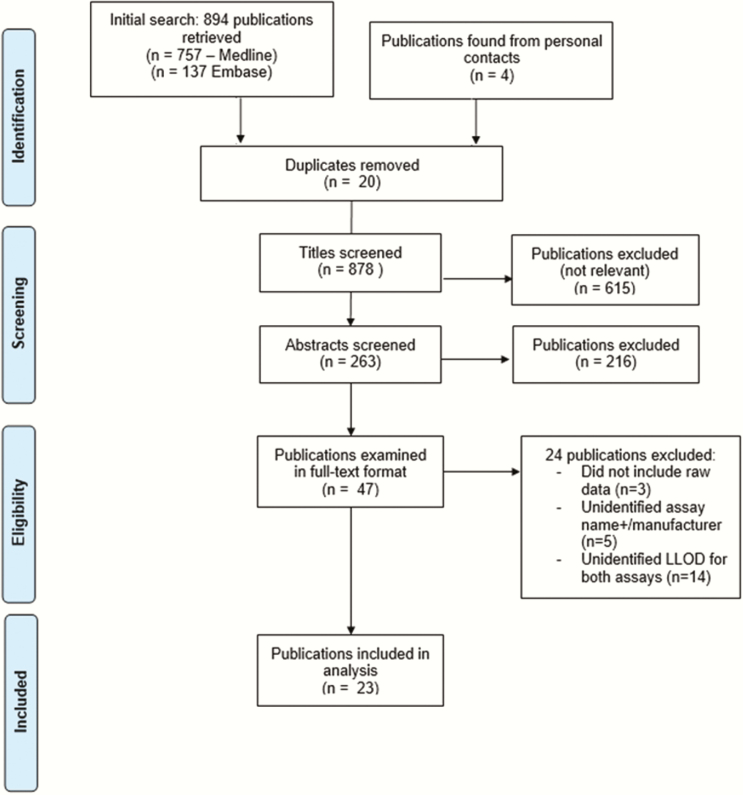
Flowchart of study selection process (PRISMA Flow Chart 2009) [3].

The review included data from 8136 samples. The number of study samples ranged from 61 to 2752 representing several populations. Eighteen studies were also from high-income countries (HIC). A flowchart demonstrating the study selection process is shown in Figure 1. A comprehensive summary of all included studies is illustrated in Supplementary Table S2.

### Percentage False Positive and False Negative

#### Antigen Versus Ribonucleic Acid (Overall)

Twenty-four datasets (representing 8136 samples) evaluated the detective accuracy of antigen assays for HCV. False-positive values ranged from 0.00% to 2.86%, and FN values ranged from 0.00% to 18.5%. Overall, 0.57% of antigen results were FP, whereas 3.52% of results were FN (Table 2). Specificity ranged from 40.00% to 100.00% and sensitivity ranged from 14.29% to 100.00% Overall, specificity was 96.63% (95% CI, 96.42–96.85) and the sensitivity was 93.94% (95% CI, 93.73–94.15).

**Table 2.** Summary of HCV Antigen and HCV RNA Detection Results

RNA Detection	Antigen Positive	Antigen Negative
RNA positive	3339 (41.04%)	286 (3.52%)
RNA negative	46 (0.57%)	4461 (54.83%)
Indeterminate	4 (0.05%)	

Abbreviations: HCV, hepatitis C virus; RNA, ribonucleic acid.

#### Architect Versus Cobas AmpliPrep/COBAS TaqMan (Group 1)

Eight datasets (representing 4427 samples) evaluated the detective accuracy of Architect cAg for HCV. False-positive values ranged from 0.00% to 1.10% and FN values ranged from 0.00% to 18.5%. Overall, 0.60% of Architect’s results were FP, whereas 2.11% of results were FN (Table 3). Specificity ranged from 95.08% to 100.00% and sensitivity ranged from to 75.83% to 100.00%. Overall specificity was 99.03% (95% CI, 98.73–99.32) and the sensitivity was 96.72% (95% CI, 96.42–97.01). A comprehensive summary of individual and grouped statistics is illustrated in Supplementary Table S3.

**Table 3.** Summary of Architect cAg (Antigen) and CATCM (RNA) Detection Results

RNA Detection	Antigen Positive	Antigen Negative
RNA positive	898 (20.57%)	92 (2.11%)
RNA negative	26 (0.60%)	3349 (76.72%)

Abbreviations: CACTM, Cobas AmpliPrep/COBAS TaqMan; cAg, core antigen; RNA, ribonucleic acid.

### Receiving Operating Charactaristic Curve

**Figure 2.**
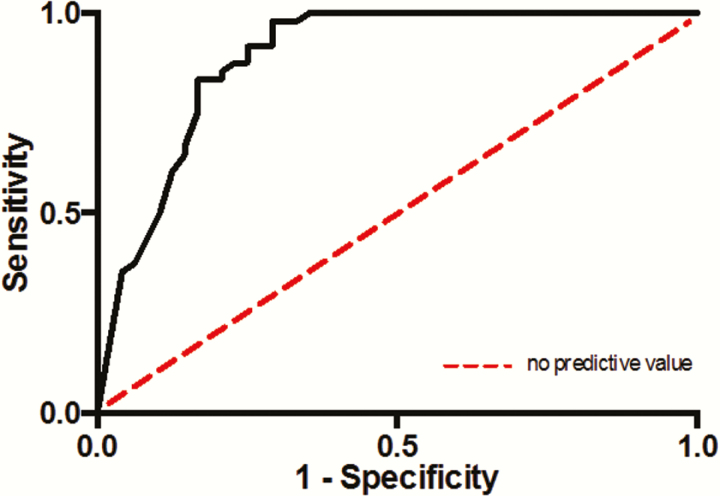
Receiver operator characteristic curve of results from all 24 included publications.

The ROC curve of all publications is shown in Figure 2. The detective accuracy of the antigen assay for HCV is illustrated by the area under the curve (AUC), which is represented by a number from 0 to 1. An area of 1 describes a perfect test (100% specificity, 100% sensitivity). When plotted for all 24 datasets, the AUC was 0.89 (95% CI, 0.82–0.96; *P* < .0001), which is statistically significant and illustrates high detective accuracy [7].

## DISCUSSION

This systematic review is the most comprehensive effort to date to examine the performance of antigen detection assays in the diagnosis of HCV. Overall, we confirm the high sensitivity and specificity of antigen assays, which, in turn, demonstrated high sensitivity and specificity for HCV. The antigen tests had low rates of FNs and FPs. Specifically, 0.57% and 3.52%, respectively, and the analysis of Architect produced an even lower FN of 2.10%.

There are some potential limitations to the review. The first is the search strategy. Although this is the largest review of its sort, only a relatively small number of papers met the inclusion criteria. At the outset, we opted not to pursue unpublished data or publications without an available English translation [8]. Furthermore, 19 studies, which did not identify the antigen used, were excluded. By having strict inclusion criteria, we hope the data included is more reliable.

Although the review captured studies from a broad range of settings, with varying degrees of HCV-burden, the results may not be applicable to low- to middle-income countries (LMIC), because 19 of 24 datasets were from HIC. Although the same assays are used internationally, additional confounders, such as genotypic variability, may exist across regions. There is increasing, albeit insufficient, evidence that genotype [9] and viral variability [10] may affect antigen and RNA assay detection results to different extents. Previous drug therapy for HCV may also act as a confounder [11], which may be important when comparing data from HIC and LMIC, because treatment availability may be very different. The priority for further data collection needs to be in high-burden/low-resource settings.

Interpretation of results needs to be cautious where RNA assays with varying LLOD were evaluated. The detective accuracy of antigen assays may be exaggerated when using a reference standard RNA with high LLOD. For example, in 5 of 6 studies that used low-sensitivity RNA assays (LLOD, 600–620 IU/mL), antigen assays had FP values of 0%–0.5%. Studies included also differed in their testing protocols with some repeating indeterminate values [12], whereas others repeated discrepant values [13]. The latter, in particular, may lead to an overexaggeration of accuracy amongst antigen assays.

Despite our general finding of a strong agreement between antigen and RNA amplification, there were isolated papers with poor sensitivity and specificity values. Poor sensitivity values of antigen assays have been underlined in various papers [14–16]—being as low as 14% [14]. These occur in many seroconverted samples after the development of HCV antibody, which can interfere with HCV-antigen detection [15, 16]. Although this interference can prevented by incubating the sample for 30 minutes with specific detergents [17], this would require additional equipment and may limit the use of HCV-antigen assays in LMIC for cases of early-infection only—because HCV antibody is detected in serology after HCV antigen [15]. A specificity value of 40% was also shown in a paper by Lorenzo et al [18]; here, only 38% of samples were within the detection range of the antigen assay used. This example underlines the disadvantages of antigen assays with higher detection limits.

More generally, the overall FN values of 3.52% are concerning, because they may mean individuals are not referred for HCV treatment. Because antigen tests have a higher LLOD than RNA tests, they are unable to detect HCV in some patients with particularly low, yet detectable, viral loads (VLs). However, there is some evidence that low-level viremia is a rare phenomenon before commencing treatment or 12 weeks after treatment completion [19]. It is possible that the sensitivity of tests could be improved by using larger samples (currently, Architect cAg uses 150 µL of plasma/serum) [20]. Although relatively less common, there are cases of FP in both analyses. The reasons for such a discrepancy are not clear, but they may include FP antigen results or FN RNA results. This may limit the use of antigen assays, because they could lead to inappropriate linkage to care and the need for further confirmatory testing.

Affordability of diagnostics is a crucial barrier to HCV management in LMIC. Cheaper diagnostics have the potential to improve access to high-quality care if this is not at the expense of diagnostic performance. The high proportion of HCV antibody-positive, RNA-negative patients means that antibody screening tests alone will not be enough to deliver high-quality care in resource-limited settings, both in terms of over investigation and treatment of patients and screening of blood products [21]. For example, studies in South Africa have illustrated a FP rate of 71% amongst discarded blood donations [22].

Although antigen tests are simpler and cheaper than RNA tests, there is room for further improvement. Currently, Architect’s assay can be performed on laboratory-based platforms, such as the i2000SR Analyzer [23]. These may be logistically difficult to implement in LMIC due to unreliable water and electricity supplies and nonadherence to storage protocols [22]. Point-of-care tests may combat this issue. For example, Daktari Diagnostics (Cambridge, MA) is developing a portable device that can perform HCV cAg detection in 30 minutes, although it is not yet commercially available [23]. Point-of-care tests may be better suited to primary healthcare facilities and could help address the need of the estimated 84% of people who do not have access to specialist laboratory facilities. However, such POC diagnostics are likely to be based on a finger-prick sample and obtaining volumes beyond 150 µL may be challenging [24].

As with human immunodeficiency virus, the use of dried-blood spots (DBS) to collect samples for HCV RNA testing could further simplify HCV management in LMIC, allowing some centralization of testing facilities [25]. The use of DBS for HCV RNA detection and quantification may be as low as 150–250 IU/mL, which is significantly lower than the currently available antigen tests but still higher than HCV RNA PCR tests [26]. This is partly due a comparatively inefficient nucleic acid extraction [27]. Nonetheless, an LLOD 150–250 IU/mL would be able to diagnose most untreated patients who present with VLs of >1000 IU/mL, but it may be inaccurate for monitoring cure [28], where viremia may be lower. There is also a poor correlation between DBS and HCV RNA concentration, hence its use would be primarily as a qualitative test. Given the introduction of directly acting antivirals, prospective HCV protocols may largely focus on qualitative for HCV diagnosis and monitoring and, therefore, could permit the widespread use of DBS in HCV management [28]. Although findings are largely positive, the questionable stability of HCV RNA in DBS may render it inferior to HCV antigen testing [28]. This could have negative implications in resource-source settings—requiring immediate transport and storage space to prevent degradation, which may not be logistically possible. Further research is necessary to confirm these variable results.

### Key Points Box

- The complexity and cost of current hepatitis C diagnostics may form a barrier to the scale-up of treatment in resource-poor settings
- Antigen tests that detect hepatitis C virus are cheaper and simpler than the current gold-standard diagnostic test (RNA tests)
- There is strong evidence that antigen detection performs as well as RNA tests and this could improve HCV care in resource-poor settings

## CONCLUSIONS

Overall, there is strong evidence for the replacement of RNA tests with antigen tests for HCV diagnosis and for the assessment of treatment response. These cheaper and simpler alternatives would have a significant impact on the HCV burden in resource-poor settings, particularly if they can be developed in technologies useable at the POC. Further efforts are needed to improve the simplicity of HCV-antigen assays to make them more suitable for implementation in LMIC. Cost-benefit analyses are also necessary to completely justify the switch to antigen tests given their FP values.

## Supplementary Data

Supplementary materials are available at *Open Forum Infectious Diseases* online. Consisting of data provided by the authors to benefit the reader, the posted materials are not copyedited and are the sole responsibility of the authors, so questions or comments should be addressed to the corresponding author.

## Supplementary Material

ofw252_suppl_1600222_Appendix
