# Supplementary material for: Can Hepatitis C Virus Antigen Testing Replace Ribonucleic Acid Polymearse Chain Reaction Analysis for Detecting Hepatitis C Virus? A Systematic Review
Source: Open Forum Infect Dis. 2017 May 26;4(2):ofw252. doi: 10.1093/ofid/ofw252 (PMC5445222; doi:10.1093/ofid/ofw252)

**Appendix**

**Table 1. Search Strategy**

| **Topic** | |
| --- | --- |
| **Search Terms** | |
| Hepatitis C | "Hepatitis C/diagnosis"[Mesh] AND |
| Assay Test | Antigen |
| **Limits** | |
| Date | 2004 – current |
| Language | English translation availability |
| **Pilot Search** | |
| Date | 2003 – current |

**Table 2: Summary of Study Characteristics**

| **Study** | **Location** | **Study Design** | **Duration** | **Sample Size** | **Population Description** |
| --- | --- | --- | --- | --- | --- |
| **Group 1** | | | | | |
| Ergunay 2011(29) | Turkey | Prospective, Cross-sectional, cohort | March 2009 – May 2010 | 272 | Hospitalised patients (various) |
| Li Cavoli 2012 (30) | Italy | Prospective, Cross-sectional, cohort | September 2009 – February 2010 | 92 | Dialysis patients |
| Miedouge 2010 (11) | France | Prospective, Cross-sectional, cohort | 2004 – 2005 (6 month period) | 2,752 | Anti-HCV negative, haemodialysis patients |
| Moscato 2011 (6) | Italy | Prospective, Cross-sectional, cohort | 5-13 month period | 61 | Previously treated, genotype 1+4 or baseline VL >200,000 IU/mL |
| Mederacke 2009 (31) | Germany | Retrospective, case-control |  | 118 | European patients with genotypes 1, 2 and 3 |
| Mederacke 2012 (32) | Germany | Retrospective, case-control |  | 237 | Different populations: HIV co-infection, hepatitis B co-infection and haemodialysis patients |
| Mixon-Hayden 2015 (33) | United States | Retrospective, case-control |  | 551 | Plasma donors and intravenous drug users |
| Park 2010 (34) | South Korea | Prospective, Cross-sectional, cohort | January – April 2009 | 282 | Suspected hepatitis C patients |
| **Group 2** | | | | | |
| Seiskari 2011 (12) | Finland | Prospective, Cross-sectional, cohort | Unspecified | 117 | Unspecified |
| Garbuglia 2014 (35) | Italy | Retrospective, case-control |  | 355 | HIV-coinfected patients |
| Shaw 2014 (36) | United Kingdom | Prospective, Cross-sectional, cohort | April 2012 – December 2013 | 112 | HIV-coinfected |
| **Group 3a** | | | | | |
| Schuttler 2004 (37) | Germany | Retrospective, case-control |  | 256 | HCV-positive, under IFN/RBV therapy |
| Alzahrani 2008 (38) | Saudi Arabia | Prospective, Cross-sectional, cohort | October 2003 – September 2006 | 297 | Intravenous drug abusers |
| Netski 2004 (39) | Thailand | Prospective, Cross-sectional, cohort | February 2009 – January 2000 | 204 | Intravenous-drug users |
| **Group 3b** | | | | | |
| Hayashi 2005 (40) | Japan | Prospective, Cross-sectional, cohort | 2002 (unspecified) | 96 | Anti-HCV positive |
| Massagua 2005 (20) | Spain | Prospective, Cross-sectional, cohort | March 2000 – August 2003 | 435 | HCV-positive patients who underwent liver transplantation for end-stage cirrhosis or HCC |
| Reddy 2006 (14) | India | Prospective, Cross-sectional, cohort | May 2003 – October 2004 | 111 | Chronic renal failure patients undergoing haemodialysis |
| Valcavi 2004 (41) | Italy | Prospective, Cross-sectional, cohort | 5 year period | 1,009 | Anti-HCV positive, hospitalised patients |
| **Group 4** | | | | | |
| Gaudy 2005 (42) | France | Prospective, Cross-sectional, cohort | December 2001 – December 2002 | 150 | Patients undergoing routine check-up |
| Soffredini 2004 (43) | Italy | Retrospective, case-control |  | 111 | Previous IFN/RBV therapy |
| **Other** | | | | | |
| Lorenzo 2004 (18) | Spain | Prospective, Cross-sectional, cohort | 2002 (unspecified) | 86 | Undergoing IFN/RBV treatment under different schedules |
| Moscato 2011 (6) | Italy | Prospective, Cross-sectional, cohort | 5-13 month period | 105 | Previously treated, genotype 1+4 or baseline VL >200,000 IU/mL |
| Buket 2014 (44) | Turkey | Prospective, Cross-sectional, cohort | September 2011 – June 2012 | 115 | Anti-HCV positive |
| Kesli 2011 (13) | Turkey | Prospective, Cross-sectional, cohort | October 2010 – April 2011 | 212 | Low-risk hepatitis C patients referred to Hospital |

**Table 3: Summary of Results (Specificity and Sensitivity) amongst 24 Datas**

| **Study** | **Antigen Test** | **LLOD (IU/mL)** | **HCV RNA Test** | **LLOD (IU/mL)** | **Total # Patient** | **Specificity (%)** | **Sensitivity (%)** |
| --- | --- | --- | --- | --- | --- | --- | --- |
| **Group 1** | | | | | | | |
| Ergunay 2011(29) | Architect HCV cAg Assay (Abbott) | <700-1100 IU/mL | COBAS AmpliPrep/ COBAS TaqMan (Roche) | <15 IU/mL | 272 | 95.08% (93.92-96.24) | 75.83% (74.79-76.86) |
| Li Cavoli 2012 (30) | Architect HCV cAg Assay (Abbott) | <700-1100 IU/mL | COBAS AmpliPrep/ COBAS TaqMan (Roche) | <15 IU/mL | 92 | 100.00% (97.06-100.00) | 90.00% (88.06-91.94) |
| Miedouge 2010 (11) | Architect HCV cAg Assay (Abbott) | <700-1100 IU/mL | COBAS AmpliPrep/ COBAS TaqMan (Roche) | <15 IU/mL | 2,752 | 99.24% (99.86-99.61) | 100.00% (99.63-100.00) |
| Moscato 2011 (6) | Architect HCV cAg Assay (Abbott) | <700-1100 IU/mL | COBAS AmpliPrep/ COBAS TaqMan (Roche) | <15 IU/mL | 61 | 100.00% (97.49-100.00) | 100.00% (97.49-100.00) |
| Mederacke 2009 (31) | Architect HCV cAg Assay (Abbott) | <700-1100 IU/mL | COBAS AmpliPrep/ COBAS TaqMan (Roche) OR Amplicor (Roche) | <15 OR <600 IU/mL | 118 | N/A | 94.92% (93.16-96.67) |
| Mederacke 2012 (32) | Architect HCV cAg Assay (Abbott) | <700-1100 IU/mL | COBAS AmpliPrep/ COBAS TaqMan (Roche) OR Amplicor (Roche) | <15 OR <600 IU/mL | 237 | 96.61% (95.36-97.86) | 98.31% (97.05-99.58) |
| Mixon-Hayden 2015 (33) | Architect HCV cAg Assay, i2000SR platform (Abbott) | <700-1100 IU/mL | COBAS AmpliPrep/ COBAS TaqMan (Roche) | <15 IU/mL | 551 | 100.00% (99.17-100.00) | 94.40% (93.59-95.21) |
| Park 2010 (34) | Architect HCV cAg Assay, i2000SR platform (Abbott) | <700-1100 IU/mL | COBAS AmpliPrep/ COBAS TaqMan (Roche) | <15 IU/mL | 282 | 100.00% (98.83-100.00) | 90.23% (89.12-91.34) |
| Weighted Average | | | | | | 99.00% (98.70-99.30) | 96.72% (96.42-97.01) |
| **Group 2** | | | | | | | |
| Seiskari 2011 (12) | Architect HCV cAg Assay (Abbott) | <700-1100 | RealTime HCV Assay, m2000sp (Abbott) | <12 | 117 | 100.00% (98.19-100.00) | 71.43% (69.90-72.96) |
| Garbuglia 2014 (35) | Architect HCV cAg Assay, i2000SR platform (Abbott) | <700-1100 | RealTime HCV Assay (Abbott) | <12 | 355 | 87.50% (86.53-88.47) | 90.48% (89.47-91.47) |
| Shaw 2014 (36) | Architect HCV cAg Assay (Abbott) | <700-1100 | RealTime HCV Assay (Abbott) | <12 | 112 | 100.00% (98.15-100.00) | 100.00% (98.15-100.00) |
| Weighted Average | | | | | | 92.40% (91.62-93.18) | 88.49% (87.72-89.25) |
| **Group 3a** | | | | | | | |
| Schuttler 2004 (37) | Trak-C (Ortho) | <15-30,000 | LightCycler RNA (Roche) | <80 | 256 | 96.23% (95.02-97.43) | 98.01% (96.80-99.22) |
| Alzahrani 2008 (38) | Trak-C (Ortho) | <15-30,000 | Cobas Amplicor V 2.0 (Roche) | <100 | 297 | 99.44% (98.31-100.00) | 93.22% (92.12-94.32) |
| Netski 2004 (39) | Trak-C (Ortho) | <15-30,000 | Cobas Amplicor V 2.0 (Roche) | <100 | 204 | 94.44% (93.34-95.55) | 86.90% (85.84-87.97) |
| Weighted Average | | | | | | 97.01% (96.31-97.71) | 93.14% (92.45-93.83) |
| **Group 3b** | | | | | | | |
| Hayashi 2005 (40) | AgIRMA (Ortho) | <15-30,000 | Amplicor 2.0 (Nippon Roche) | <500 | 96 | 100.00% (98.00-100.00) | 96.74% (94.77-98.71) |
| Massagua 2005 (20) | Trak-C (Ortho) | <15-30,000 | Cobas Amplicor V 2.0 (Roche) | <600 | 435 | 100.00% (99.06-100.00) | 92.20% (91.29-93.10) |
| Reddy 2006 (14) | (Ortho) | <15-30,000 | Cobas Amplicor V 2.0 (Roche) | <600 | 111 | 97.78% (95.95-99.62) | 14.29% (13.58-14.99) |
| Valcavi 2004 (41) | Trak-C (Ortho) | <15-30,000 | Cobas Amplicor V 2.0 (Roche) | <600 | 1,009 | 99.51% (98.89-100.00) | 94.70% (94.10-95.30) |
| Weighted Average | | | | | | 99.55% (99.07-100.00) | 88.75% (88.39-89.21) |
| **Group 4** | | | | | | | |
| Gaudy 2005 (42) | Trak-C (Ortho) | <15-30,000 | Versant HCV 3.0 (Bayer) | <620 | 150 | 100.00% (98.40-100.00) | 96.69% (95.12-98.27) |
| Soffredini 2004 (43) | Trak-C (Ortho) | <15-30,000 | Versant HCV 3.0 (Bayer) | <615 | 111 | 100.00% (98.14-100.00) | 94.55% (92.74-96.35) |
| Weighted Average | | | | | | 100.00% (98.79-100.00) | 95.78% (94.59-96.96) |
| **Other** | | | | | | | |
| Lorenzo 2004 (18) | Trak-C (Ortho) | <15-30,000 | Cobas Amplicor V 2.0 (Roche) | <250 | 86 | 40.00% (38.66-41.34) | 87.65% (85.68-89.63) |
| Moscato 2011 (6) | Architect Core Ag (Abbott) | <700-1100 | Versant HCV 3.0 (Bayer) | <615 | 105 | 0.00% (0.00-0.00) | 100.00% (98.09-100.00) |
| Buket 2014 (44) | Architect Core Ag, i2000SR (Abbott) | <700-1100 | RT-PCR (Anatolia) | <25 | 115 | 100.00% (98.17-100.00) | 86.46% (84.76-88.16) |
| Kesli 2011 (13) | Architect Core Ag, i2000SR (Abbott) | <700-1100 | Artus HCV (Qiagen) | <20 | 212 | 100.00% (98.65-100.00) | 96.25% (94.93-97.57) |
| **OVERALL WEIGHTED AVERAGE** | | | | | | **96.59% (96.37-96.80)** | **93.96% (93.75-94.17)** |

**Figure 1: QUADAS 2 Graph: Risk of Bias and Applicability**

**
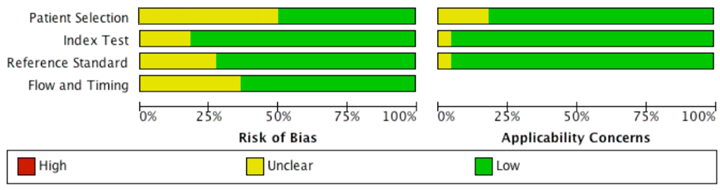
**


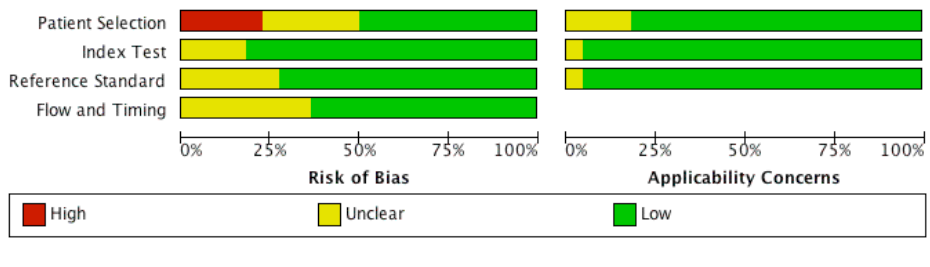


**Figure 2: QUADAS 2 Summary: Risk of Bias and Applicability**


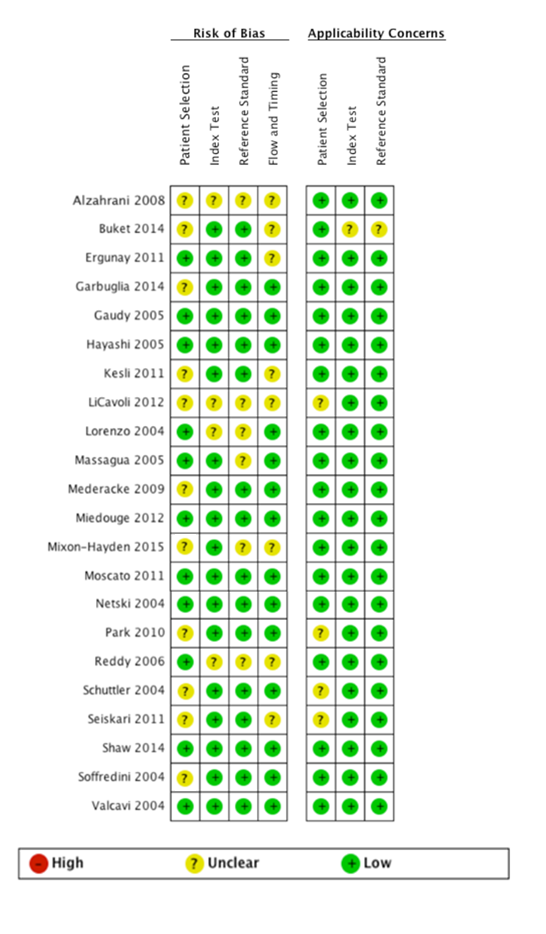

Supplement: ofw252_suppl_1600222_Appendix [file ofw252_suppl_1600222_appendix.docx]
